# “STOP HASSLING ME!”: HASSLES, PERCEIVED SUPPORT, AND MENTAL HEALTH OUTCOMES IN OLDER ADULTHOOD

**DOI:** 10.1093/geroni/igac059.2638

**Published:** 2022-12-20

**Authors:** Amy Gourley, Lucas Hamilton, Max Coleman, Anne Krendl

**Affiliations:** Indiana University, Bloomington, Indiana, United States; Indiana University, Bloomington, Indiana, United States; Indiana University, Bloomington, Indiana, United States; Indiana University, Bloomington, Indiana, United States

## Abstract

Prior work suggests that older adults with larger social networks have better mental health, potentially due to having greater access to support from different network members. However, other research suggests that perceptions of support, which do not necessarily align with network size, predict better mental health and higher life satisfaction. It could be that, irrespective of network size, the presence of hassles – network members that are perceived as causing problems or making life difficult – is associated with lower perceptions of support, which negatively impacts mental health. We tested this possibility using social network data from 137 older adults (Mage = 74.25 years) who completed the PhenX social interview and well-validated measures of depression, perceived social support, and life satisfaction. In linear regression models controlling for other network variables, perceived support and the level of hassles independently predicted depression and life satisfaction. Testing our mediation predictions, hassles significantly mediated the effect of perceived support on depression and life satisfaction. Alternatively, perceived support only mediated the effect of hassles on life satisfaction. Taken together, these findings may indicate that the presence of hassles in one’s network predicts mental health symptomology and life satisfaction, irrespective of perceptions of support or other network characteristics (e.g., network size). Thus, while perceptions of social support appear to be important predictors of life satisfaction, network characteristics better predict mental health symptomology. Future work on social engagement interventions may benefit from a targeted approach depending on the outcome of interest.

